# Measuring caregiver activation to identify coaching and support needs: Extending MYLOH to advanced chronic illness

**DOI:** 10.1371/journal.pone.0205153

**Published:** 2018-10-11

**Authors:** Soo Borson, Patrick Mobley, Karl Fernstrom, Paige Bingham, Tatiana Sadak, Heather R. Britt

**Affiliations:** 1 Psychiatry and Behavioral Sciences, University of Washington School of Medicine and Psychosocial and Community Health, School of Nursing, Seattle, Washington; 2 Data Scientist, Boston, Massachusetts; 3 Research Scientist, Health Policy and Health Economics, Minnesota Department of Health, St. Paul, Minnesota; 4 Director, Allina Health Group, Minneapolis, Minnesota; 5 Psychosocial and Community Health, University of Washington School of Nursing, Seattle, Washington; 6 Senior Director, Minnesota Hospital Association, St. Paul, Minnesota; University of Michigan Medical School, UNITED STATES

## Abstract

**Introduction:**

Family and friends of seriously ill patients are key partners in providing support and health care at home, managing relationships with clinicians, and navigating complex health care systems. Becoming a knowledgeable, confident, and effective caregiver is a developmental process we term 'caregiver activation' and could be facilitated by clinicians equipped with suitable tools. Managing Your Loved One’s Health (MYLOH) is a new tool to identify gaps in caregivers' knowledge, skills, and access to clinical and personal support. Created in partnership with caregivers and clinicians, MYLOH items reflect the essential dimensions of caregiving and can be used to tailor caregiver coaching to domains of greatest need. In this study, we extend MYLOH's initial focus on dementia care to caregivers of patients with other chronic life-limiting illnesses.

**Methods:**

MYLOH was completed by primary caregivers (n = 190) of people with a range of advanced chronic illnesses enrolled in the LifeCourse study, an innovative, whole-person approach to health management. Item relevance and responses were compared by group across MYLOH items and domains using z-tests for equality of proportions.

**Results:**

All MYLOH items were relevant to caregiving for all types of chronic illness; only 13% of caregivers answered “not my responsibility” to any question. MYLOH identified caregiving struggles across patient diagnosis groups with a few, disease-specific ‘hotspots’. Overall, 64% of caregivers scored low in activation on at least one healthcare management task, especially getting enough help with caregiving, managing everyday caregiving tasks, understanding/managing medications, and knowing how to respond to rapid changes in care recipients' health status. No difficulty was unique to a specific type of care recipient illness.

**Conclusions:**

MYLOH has potential as a tool for identifying caregiver coaching and support needs in managing a range of serious chronic illnesses. Caregiving difficulties endorsed by over 20% of caregivers should be core components of chronic illness management programs regardless of disease focus, with disease-specific tailoring as required. MYLOH may be useful in evaluating caregiver interventions and health systems’ performance in integrating caregivers into the care management of patients with complex life-limiting illness.

## Introduction

Family and friends are essential clinical partners in managing the health care of chronically ill older people [1], yet formal engagement, training, and support for their critical roles has been slow to develop [2]. Failure to engage, teach, and support caregivers may contribute to poor patient outcomes, including potentially preventable patient hospitalizations [3,4,5], and negatively affect caregiver health and cognitive function [6,7]. Managing Your Loved One’s Health (MYLOH) is a new valid and reliable caregiver-centered tool that measures self-rated knowledge and skill across a core set of health care domains agreed on by both clinicians and caregivers as integral to the caregiving role [5,8,9]. MYLOH scores measure the level of ‘caregiver activation’ for health care overall and across specific domains of knowledge and skill: understanding and managing patients’ everyday symptoms and worrisome changes in health, medications, engaging providers, advocating for the patient in health care situations, and caring for one’s own needs as a caregiver. MYLOH was developed to help guide conversations around health care management of medically complex patients with dementia and identify needs for targeted caregiver coaching, but its domains encompass aspects of care that are common to chronic diseases generally [10].

Previous research using MYLOH to measure caregiver activation has focused on caregivers for individuals with dementia. The present study is the first to examine activation among caregivers of older people with other serious chronic conditions as well as those with advanced dementia. Our primary goals are twofold: (1) to examine commonalities and distinctive challenges encountered by caregivers as a function of the patient’s primary diagnosis and (2) to define specific domains of risk for suboptimal health care management based on caregivers’ self-reports. Results should inform the design of coaching interventions for caregivers as part of the health care of patients with chronic illness.

## Methods

### Study background and context

This study was approved by Quorum institutional review board (protocol number 28142/1). This is a descriptive study of self-identified primary caregivers (n = 190) of people with serious chronic illness (primarily cancer, dementia, and heart failure) enrolled in the LifeCourse program at Allina Health, a large Midwest healthcare system [11]. LifeCourse is a specialized, person-centered care management program for patients with life-limiting illness [12]. Participants in the present study are part of the initial pilot project from which a clinical expansion is planned. Between October 2012 and June 2016, 1018 potentially eligible caregivers had been identified. Due to deployment of MYLOH mid-study in the LifeCourse project, baseline data were available for 471 caregivers, including 203 self-identified key friends and family. After excluding 13 with >20% of MYLOH items scored ‘missing’ or ‘not my responsibility’, 190 participants were considered primary caregivers with responsibility for health care oversight and comprised the final sample for analysis. Of these, 172 responded to all 29 items; for the 18 with any missing data, the average number of skipped items was 2. Missing responses did not count towards the total in calculating and testing proportions for that item.

### Patient characteristics

Patients (care recipients) of enrolled caregivers were older adults (mean age = 77 years) with cancer (n = 29), dementia (n = 76), or other serious chronic disease (n = 85) which included heart failure [n = 69], COPD [n = 10], aortic valve disease [n = 4], and end-stage renal disease [n = 2]. Care recipients were selected in a step-wise process starting with electronic health record search criteria that included emergency department and inpatient utilization, a life-limiting diagnosis verified by individual medical record review by an experienced registered nurse, and a specified minimum combined Charlson-Elixhauser comorbidity score [13]. This score combines two widely-used comorbidity measures, each counting specific diseases assigned an empirically derived prognostic weight, into a single score that is generated algorithmically from administrative data using ICD9/10 diagnoses. Care recipients with a primary diagnosis of dementia had no specified minimum comorbidity score while all others had to have a minimum score of 4, consistent with a greater than 80% probability of surviving at least one year [13]. While there is no 'typical' patient with a given score, an individual scoring 4 on the combined index might have congestive heart failure, diabetes with complications, and peripheral vascular disease, or chronic obstructive pulmonary disease, a cardiac arrhythmia, and weight loss. Care recipients did not meet criteria for hospice (≤ 6 months life expectancy) at the time of LifeCourse enrollment, but were sick enough to benefit from a supportive care approach. In LifeCourse, care recipients and their participating family caregivers are surveyed at baseline and thereafter every 3 months. Here we report baseline demographic and caregiver activation data.

### Caregiver activation measure: MYLOH

MYLOH’s 29 items are grouped into 7 domains: understanding and managing patients’ everyday problems (with cognition, mood and behavior, and health), recognizing and reacting to rapidly worsening changes in health, understanding and managing medications, making health care decisions, and getting help and support for oneself as a caregiver. Items are structured as self-report statements in a 4-level Likert type response format (4 = agree completely, 3 = agree, 2 = disagree, 1 = disagree completely) plus a 0 option ('not my job') to account for variation in caregiver types and roles. Based on test theory and extensive feedback from clinicians and caregivers in the initial measure development phase, no neutral option is offered [8]. MYLOH is intended as a tool to identify caregivers who may be having difficulty managing specific aspects of the health care of an older adult and sustaining their own wellbeing. Scoring 'disagree' or 'disagree completely' on any item indicates at least some difficulty (low activation) with respect to that specific element of caregiving. We interpret such responses as indicating a potential need for tailored caregiver coaching, monitoring, and follow up.

#### Analysis

We grouped “disagree” and “disagree completely” responses into a single identifier of need for coaching, support, and/or follow-up (low activation). The percentage of these ‘need’ responses were tabulated by item; respondents with at least one were considered positive for need in the domain containing that item. We used z-tests of the equality of proportions to compare caregivers across each care recipient diagnosis group (cancer, dementia, and other) to the other two. In addition, we examined the percentage of “not my responsibility” responses to index the relevance of items and domains to caregiving roles across diagnosis groups.

## Results

### Sample description

Caregivers in all three illness groups were white (99%) and most were over age 60 (Table 1). Caregivers for patients with serious chronic illnesses other than dementia and cancer were nearly all spouses/domestic partners (92%). Caregivers of dementia patients were less often spouses (49%), and more often adult sons or daughters. Caregivers of cancer patients were predominantly spouses (79%), less often women (55%) and generally more educated than the other groups.

**Table 1:** Characteristics of caregivers and care recipients by diagnosis group.

Caregiver	Cancer (n = 29)	Dementia (n = 76)	Other Diseases* (n = 85)
Age (mean ± sd)	63 ± 12	67 ± 13	64 ± 13
Married or living with partner	93%	74%	86%
Female	55%	74%	75%
White	100%	100%	99%
Lives with Patient	93%	62%	73%
Education			
HS or less	17%	22%	21%
Some college to bachelor's	48%	50%	55%
Grad/professional school	34%	26%	22%
Unknown	0%	1%	1%
Relation to care recipient			
Spouse/Partner	79%	49%	92%
Son/Daughter	14%	42%	4%
Brother/Sister	3%	0%	2%
Father/Mother	3%	1%	1%
Other	0%	8%	0%
Contact frequency with care recipient			
Daily	100%	89%	93%
Weekly	0%	9%	7%
Monthly	0%	1%	0%
**Care Recipient**			
Age (mean ± sd)	66 ± 13	83 ± 8	75 ± 12
Comorbidity (mean ± sd)	5 ± 1	2 ± 2	5 ± 1
Married or living with partner	79%	53%	64%
Female	48%	39%	40%
White	100%	96%	96%
Education			
HS or less	14%	42%	28%
Some college to bachelor's	48%	39%	53%
Grad/professional school	34%	18%	14%
Unknown	3%	0%	5%
Location			
Lives at Home	100%	76%	87%
Nursing Home	0%	3%	2%
Assisted Living	0%	21%	9%

* "Other" includes congestive heart failure, aortic valve disease, chronic obstructive pulmonary disease, and chronic kidney disease.

Care recipient groups varied more by age (mean ages: cancer = 66, dementia = 83, other = 75), and were also mostly white (97%). Of the three care recipient groups, the dementia group had the lowest mean comorbidity score (1.6 ± 1.9) and fewest women (39%), were generally less educated and more often living in paid residential care (21%) than the other two groups. Dementia patients were least likely to live with the primary caregiver (62%), but 90% of caregivers still maintained daily contact. Cancer patients lived at home (100%) and about half (48%) were women.

### Item relevance to caregiving roles

87% of caregivers answered all items with a score of 1–4, indicating that all questions were relevant to their caregiving role; only 13% answered “not my responsibility” to any question. In one notable item (Q13), 7% of caregivers overall (12% of caregivers for people with non-cancer, non- dementia diagnoses and 3% for caregivers of people with cancer or dementia) responded “not my responsibility” to the statement: “I know how much help he/she needs from me to take medications as recommended by a healthcare provider.” This group difference was not explained by any demographic characteristic of caregivers or recipients (S1 Table).

### Caregiving “Hotspots”: Key domains

MYLOH identified caregiving struggles across all three patient diagnosis groups (Table 2): 64% of all caregivers scored low in activation (i.e., in need of coaching, support, or follow-up) on at least one specific health care management task. Every domain, in every diagnosis group, contained caregivers needing additional support—suggesting that MYLOH identifies caregiving problems across a range of chronic conditions and is relevant to caregivers for other chronic illnesses as well as dementia. Ordered by total percentages, Table 2 shows domains with which caregivers commonly have difficulty. All groups have problems with getting enough support (38.9% overall; proportions were highest for dementia caregivers at 46.1% (p = 0.10) and for most, managing care recipients’ everyday problems were their next most common concern (31.1%). One quarter of all caregivers and nearly a third of caregivers for patients with non-cancer, non-CNS diseases (p = 0.06) reported concerns around medication management, and similar proportions were unsure how to react and what to do when a patient’s health status rapidly worsened. Making healthcare decisions (including legal aspects, advocacy for the patient, and knowing what matters most to him or her) was the domain least often identified as a problem for caregivers. Of the three caregiver groups, dementia caregivers reported the widest range of responses across caregiving domains: 41% (p = 0.02) reported high need for help managing day-to-day problems and 4% (p = 0.05) reported low need around making health care decisions. Notably, caregivers of patients with non-cancer, non-CNS diagnoses had more difficulty with medication management but less with managing day-to-day problems, compared to caregivers of patients with dementia or cancer.

**Table 2:** Percent, by domain, of caregivers with at least one "low activation" response, suggesting a need for coaching, support, and follow-up.

Domain	Total (n = 190)	Cancer (n = 29)		Dementia (n = 76)		Other Diseases (n = 85)	
	%	%	P—value	%	P—value	%	P—value
Support for caregiving	38.9	27.6	0.17	46.1	0.10	36.5	0.53
Managing patients’ day-to-day problems	31.1	27.6	0.66	40.8	0.02	23.5	0.04
Medication aspects	26.3	20.7	0.46	21.1	0.18	32.9	0.06
Reacting to rapidly worsening changes	23.7	24.1	0.95	26.3	0.49	21.2	0.46
Understanding patients’ problems	16.3	10.3	0.34	18.4	0.52	16.5	0.96
Recognizing rapidly worsening changes	16.3	6.9	0.40	21.1	0.15	15.3	0.73
Making healthcare decisions	8.9	13.8	0.32	3.9	0.05	11.8	0.22

A test of the equality of proportions (z-test) was used to compare caregivers across each care recipient diagnosis group (cancer, dementia, and other) to the combined other two.

### Item-level caregiving problems

For most items (Table 3), caregivers across patient diagnostic groups expressed similar needs for more caregiving support; nearly two thirds of MYLOH items (18/29) yielded no significant diagnosis-related differences. The single item most often identified as a problem (27% of all caregivers combined) was Q28: “I know how to get help with day-to-day caregiving when needed”. This item was also the most common concern (32.9%, p = 0.13) for dementia caregivers. That means that nearly a third of all caregivers in our sample struggle to find enough help for day-to-day caregiving tasks.

**Table 3:** Percent of individuals with a response suggesting a need for additional coaching, support, or follow-up, by item.

Item	Total	Cancer		Dementia		Other	
	(n = 190)	(n = 29)		(n = 76)		(n = 85)	
	%	%	P	%	P	%	P
**Support for caregiving**							
Help with day-to-day tasks (Q28)	27.0	13.8	0.08	32.9	0.13	26.2	0.83
Help with care during personal emergency (Q29)	25.0	20.7	0.56	29.3	0.26	22.6	0.50
Ability to provide all needed care (Q26)	14.3	3.4	0.07	19.7	0.08	13.1	0.68
Taking care of myself (Q27)	13.3	6.9	0.27	18.4	0.09	10.8	0.38
**Managing patients’ day-to-day problems**							
With self-care (Q8)	20.3	3.4	0.01	32.0	0.00	15.7	0.16
With mood/behaviors (Q6)	20.2	17.9	0.74	27.6	0.04	14.3	0.07
With physical health (Q7)	15.4	13.8	0.79	17.3	0.56	14.3	0.70
With memory (Q5)	10.1	0.0	0.05	14.5	0.10	9.6	0.85
**Understanding patient medications**							
Positive and negative expectations (Q12)	21.9	10.3	0.10	17.6	0.24	29.8	0.02
Dosage (Q10)	10.1	13.8	0.47	6.7	0.20	11.9	0.46
When and how to use (Q11)	10.1	10.3	0.96	8.0	0.44	11.9	0.46
How much help care recipient needs (Q13)	6.3	3.4	0.49	4.0	0.28	9.4	0.12
Understanding provider recommendations (Q9)	5.8	6.9	0.79	1.3	0.03	9.4	0.06
**Reacting to rapidly worsening changes**							
Which provider to contact (Q21)	14.2	20.7	0.28	13.2	0.73	12.9	0.65
What to report/watch for (Q18)	13.8	17.2	0.55	12.0	0.57	14.1	0.90
What I can manage on my own (Q19)	11.6	6.9	0.39	13.3	0.56	11.8	0.96
When to contact the provider (Q20)	9.5	17.2	0.12	7.9	0.54	8.2	0.60
When to call 911 (Q22)	4.8	6.9	0.56	5.3	0.79	3.6	0.49
**Understanding patients’ problems**							
With mood/behaviors (Q2)	10.6	6.9	0.48	9.2	0.60	13.3	0.30
With self-care (Q4)	7.9	6.9	0.82	9.2	0.60	7.1	0.72

A test of the equality of proportions (z-test) was used to compare caregivers across each care recipient diagnosis group (cancer, dementia, and other) with the combined other two.

Our data also reveal some unique challenges for caregivers based on diagnosis group. Not surprisingly, dementia caregivers had particular challenges managing care recipients' problems with self-care (Q8, 32.0%, p = 0.001) and mood/behaviors (Q6, 27.6%, p = 0.04). These two items (Q8 & Q6) were more often scored as problematic by dementia caregivers than others in our sample (denoted in Table 3). Among caregivers of patients with other chronic serious illness, nearly a third–more than those caring for individuals with cancer or dementia–indicated that they did not understand what to expect from patients’ medications (Q12, 29.8%, p = 0.02).

## Discussion

MYLOH appears to have good potential for identifying caregivers' needs for coaching and support that are common across diverse serious illnesses. These concerns span several different aspects of caregiving—especially finding enough help with caregiving, managing day-to-day tasks, understanding and managing medications, and knowing how to respond to rapid changes in patients' health. These four domains, problematic for at least 20% of caregivers in all groups, are therefore essential components of any caregiver needs assessment and support plan, regardless of patient diagnosis group.

Our study also identifies specific health care management tasks that–though not unique to any caregiver group–are more likely to pose problems for one than another. These include understanding what to expect from medications (more often a problem for caregivers of patients with heart failure and other serious systemic diseases, whose medication regimens may be highly complex and/or poorly explained by clinicians) and how to manage self-care deficits and mood and behavior changes (more often a problem for, but not limited to, dementia caregivers). Group differences across caregiving domains likely reflect complex interactions between the changing health of the patient, evolving caregiving demands, the trajectory of the caregiving ‘career’, and individual characteristics of caregivers including adaptive developmental processes that occur in response to experience. Previous work with MYLOH indicates that the tool can reflect such developmental evolution and suggesting its usefulness in working with caregivers over time [8].

### Limitations

Some limitations of this descriptive study are the small sample size in total and dissimilar sample sizes between patient diagnosis groups in the tests of proportions. Group differences in caregiver demographics, rather than differences in caregiving experience based on the needs of different patient groups, might explain some of the differences in activation item/domain scores; our sample size is too small to fully address this possibility. Other limitations include low representation of racial and ethnic minorities, consistent with population demographics of Minnesota generally and Allina Health in particular, and individuals younger than age 60.

One item (Q13, understanding care recipient’s need for help with medication management) yielded a relatively high prevalence of “not my responsibility” responses from caregivers of patients with major non-cancer, non-CNS diseases. Three explanations are possible: the care recipient manages his/her own medications independently and the caregiver has no concerns about this; or someone other than the primary caregiver or patient has this responsibility. Our data do not distinguish among these possibilities. We did not observe many 'not my responsibility' answers in previous studies of MYLOH, which were limited to dementia caregivers [8,9,14]. Further investigation of the underlying reasons for this response variability is warranted.

Finally, we did not investigate whether duration of caregiving influenced responses. Our previous work suggests that MYLOH responses can reflect the developmental evolution of caregiving activation, linked to the accumulation of caregiving knowledge and skill with experience [8].

## Conclusions

“Managing Your Loved One’s Health” (MYLOH) was designed to guide conversations between clinicians and caregivers around need for coaching and support in caring for patients with chronic illness at home. This study shows that MYLOH is a promising tool for health care delivery research on family caregiving for patients with diverse chronic illness and for design of testable caregiver interventions to support their role in health care management. Further research should evaluate the value and clinical utility of MYLOH as a broad-spectrum outcome measure in complex chronic care management. Caregiver activation reflects a distinct construct that is positively related to caregiver mental wellbeing and preparedness for caregiving, and negatively related to caregiver stress and anxiety [8]. Additional research efforts should address how caregiver activation may relate to actual capacity and health of caregivers and to patient outcomes.

## Supporting information

S1 Table Percent of individuals with a “not my responsibility” response by item. (DOCX)
