# Supplementary material for: Measuring caregiver activation to identify coaching and support needs: Extending MYLOH to advanced chronic illness
Source: PLoS One. 2018 Oct 11;13(10):e0205153. doi: 10.1371/journal.pone.0205153 (PMC6181336; doi:10.1371/journal.pone.0205153)
Supplement: S1 Table — (DOCX) [file pone.0205153.s001.docx]

**S1 Table. Percent of individuals with a “Not My Responsibility” response by item.**

| **Item** | **Total** | **Cancer** | **Dementia** | **Other** |
| --- | --- | --- | --- | --- |
|  | **(n = 190)** | **(n = 29)** | **(n = 76)** | **(n = 85)** |
| **Medication management** |  |  |  |  |
| How much help they need (Q13) | 6.9% | 3. 5% | 2.7% | 11.8% |
| Dosage (Q10) | 3.2% | 3.5% | 1.3% | 4.8% |
| When and how to use (Q11) | 3.2% | 3.5% | 1.3% | 4.8% |
| Understanding provider recommendations (Q9) | 2.7% | 3.5% | 1.3% | 3.5% |
| Positive and negative expectations (Q12) | 2.1% | 3.5% | 1.4% | 2.4% |
| **Managing patients’ day-to-day problems** |  |  |  |  |
| With self-care (Q8) | 2.1% | 0.0% | 2.7% | 2.4% |
| With memory (Q5) | 1.6% | 0.0% | 0.0% | 3.6% |
| With mood/behaviors (Q6) | 0.5% | 0.0% | 0.0% | 1.2% |
| With physical health (Q7) | 0.5% | 0.0% | 0.0% | 1.2% |
| **Making healthcare decisions** |  |  |  |  |
| Power of Attorney responsibilities (Q25) | 1.6% | 3. 5% | 0.0% | 2.4% |
| What is important to care recipient (Q23) | 0.0% | 0.0% | 0.0% | 0.0% |
| Care recipient advocacy (Q24) | 0.0% | 0.0% | 0.0% | 0.0% |
| **Understanding patients’ problems** |  |  |  |  |
| With memory (Q1) | 1.1% | 0.0% | 0.0% | 2.4% |
| With mood/behaviors (Q2) | 1.1% | 0.0% | 0.0% | 2.4% |
| With self-care (Q4) | 1.1% | 3.5% | 0.0% | 1.2% |
| With physical health (Q3) | 0.5% | 0.0% | 0.0% | 1.2% |
| **Support for caregiving** |  |  |  |  |
| Ability to provide all needed care (Q26) | 1.1% | 0.0% | 0.0% | 2.4% |
| Help with day-to-day tasks (Q28) | 1.1% | 0.0% | 1.3% | 1.2% |
| Taking care of myself (Q27) | 0.0% | 0.0% | 0.0% | 0.0% |
| Help with care during personal emergency (Q29) | 0.0% | 0.0% | 0.0% | 0.0% |
| **Reacting to rapidly worsening changes** |  |  |  |  |
| What I can manage on my own (Q19) | 0.5% | 0.0% | 0.0% | 1.2% |
| When to call 911 (Q22) | 0.5% | 0.0% | 1.3% | 0.0% |
| What to report/watch for (Q18) | 0.0% | 0.0% | 0.0% | 0.0% |
| When to contact the provider (Q20) | 0.0% | 0.0% | 0.0% | 0.0% |
| Which provider to contact (Q21) | 0.0% | 0.0% | 0.0% | 0.0% |
| **Recognizing rapidly worsening changes** |  |  |  |  |
| With mood/behavior (Q15) | 0.5% | 0.0% | 0.0% | 1.2% |
| With self-care (Q17) | 0.5% | 0.0% | 0.0% | 1.2% |
| With memory (Q14) | 0.0% | 0.0% | 0.0% | 0.0% |
| With physical health (Q16) | 0.0% | 0.0% | 0.0% | 0.0% |

Tests of equality of proportions were not performed on these data.
